# Rudiments of Modern Medical Electricity

**Published:** 1902-09

**Authors:** 


RUDIMENTS OF MODERN MEDICAL ELECTRICITY. Arranged in the form of questions and answers. Prepared especially for students of medicine. By S. H. Monell, M. D., New York, Professor of Static Electricity in the International Correspondence School, etc. Pages, 165. Price, $1.00. New York: Edward R. Pelton, Publisher, 19 East 16th St. 1900.

This little book does not seem to be of much value to the general practitioner, but to the author it has possibly amounted to something. As stated in the title, the subject matter is arranged in the form of questions and answers, but they all lead to author's larger works. T. J. B.

